# Geographical Differentiation and Quality Evaluation of *Lonicerae japonicae* Flos by Integrating HS–GC–IMS Volatile Profiling with In Vivo Pharmacodynamic Assessment

**DOI:** 10.3390/foods15183305

**Published:** 2026-09-18

**Authors:** Yanghai Wang, Ruijun Lyu, Hongchao Shang, Yuchen Yao, Bonian Zhao, Yan Gao, Lu Liu

**Affiliations:** 1 Institute of Pharmacy, Shandong University of Traditional Chinese Medicine, Jinan 250355, China; 2024100159@sdutcm.edu.cn (Y.W.); 60011983@sdutcm.edu.cn (B.Z.); 2 Shandong Key Laboratory of Digital Traditional Chinese Medicine, Shandong University of Traditional Chinese Medicine, Jinan 250355, China

**Keywords:** *Lonicerae japonicae* Flos, geographical differentiation, HS–GC–IMS, volatile organic compounds, OPLS–DA, secondary bacterial infection

## Abstract

*Lonicerae japonicae* Flos (LJF) is a medicinal and edible herbal material whose chemical characteristics and biological activity may vary with geographical origin. In this study, 27 LJF batches from Shandong, Henan, and Hebei Provinces were evaluated using headspace gas chromatography–ion mobility spectrometry (HS–GC–IMS), multivariate analysis, and a mouse model of *Staphylococcus aureus* superinfection following influenza A (H1N1) virus infection. HS–GC–IMS detected 62 volatile ion signals; 49 were tentatively assigned to 36 unique volatile organic compounds (VOCs), while 13 remained unidentified. Fingerprint analysis revealed distinct origin-associated differences in volatile profiles, and OPLS–DA showed the clearest separation of Shandong samples from those from Henan and Hebei (R^2^Y = 0.898; Q^2^ = 0.844). Thirty-two VIP-ranked signals were identified as candidate discriminant variables. In vivo, LJF aqueous extracts reduced the lung index, pulmonary bacterial burden, and relative NP RNA expression. Samples from Shandong and Henan generally showed greater improvements than those from Hebei across the evaluated endpoints. Overall, both the chemical and pharmacodynamic datasets indicated origin-associated variation in LJF. Direct relationships between specific volatile constituents and pharmacodynamic effects, however, were not established. These findings support the integration of HS–GC–IMS profiling with pharmacodynamic assessment as a practical framework for origin-related quality evaluation of LJF, while targeted confirmation of key discriminatory VOCs and further chemical–efficacy analyses remain warranted.

## 1. Introduction

*Lonicerae japonicae* Flos (LJF), the dried flower buds or newly opened flowers of *Lonicera japonica* Thunb. (Caprifoliaceae), is a traditional medicinal and edible herbal material widely used in China. It is produced mainly in Shandong, Hebei, and Henan Provinces, which are recognized as major production areas [1,2]. LJF has been reported to exhibit antiviral [3], antibacterial [4], and anti-inflammatory activities [5] and is used in pharmaceutical preparations, functional foods, and personal care products.

The chemical composition of LJF is influenced by environmental and agronomic conditions associated with geographical origin. Samples from different regions can vary markedly in the composition and abundance of volatile constituents [6], potentially contributing to differences in chemical characteristics and biological activity. Pharmacopoeial quality control and geographical authentication, however, serve different analytical purposes. The quantitative markers specified in the Pharmacopoeia of the People’s Republic of China—chlorogenic acid, 3,5-O-dicaffeoylquinic acid, 4,5-O-dicaffeoylquinic acid, and luteoloside—support identity and quality consistency, but their combined contents may have limited discriminatory power among production regions [7]. Complementary analytical information that is sensitive to geographical origin is therefore required when regional differentiation is the specific objective.

Headspace gas chromatography–ion mobility spectrometry (HS–GC–IMS) combines chromatographic separation with sensitive ion-mobility detection and has been increasingly applied to food traceability and the geographical differentiation of herbal materials [8,9,10]. Rapid analysis and minimal sample preparation are advantageous for volatile profiling because extensive handling can alter headspace composition. However, HS–GC–IMS assignments are generally tentative when authentic standards are unavailable, and signal patterns may be affected by storage history, equilibration conditions, and other headspace parameters. Standardized sample handling and analytical conditions are therefore essential for reliable origin-related comparisons.

For medicinal and edible herbal materials, origin-related chemical differences are more informative when considered alongside biological performance. Our previous studies showed that LJF exhibited activity against influenza A (H1N1) virus infection [11,12] and *Staphylococcus aureus* infection [13]. On this basis, we examined whether geographical differences in volatile profiles were accompanied by corresponding differences in protective activity in a mouse model of *S. aureus* superinfection following influenza A virus infection.

Accordingly, the volatile profiles of 27 LJF batches collected from Shandong, Henan, and Hebei Provinces were characterized by HS–GC–IMS and evaluated using multivariate analysis. Aqueous extracts prepared from the same batches were then compared in the superinfection model. By examining chemical and pharmacodynamic variation within the same sample set, this study aimed to provide a more comprehensive basis for origin-related quality evaluation of LJF.

## 2. Materials and Methods

### 2.1. Materials and Reagents

Detailed information on sample codes, collection locations, and harvest information is provided in Supplementary Table S1. All LJF samples were oven-dried after harvest and subsequently stored until analysis.

Influenza A virus A/PR/8/34 (H1N1; PR8 strain) was provided by the Antiviral Research Center of Shandong University of Traditional Chinese Medicine (Jinan, Shandong, China). *Staphylococcus aureus* ATCC 6538 was obtained from the American Type Culture Collection (Manassas, VA, USA). Oseltamivir phosphate granules were purchased from Yichang HEC Changjiang Pharmaceutical Co., Ltd. (Yidu, Hubei, China), and phosphate-buffered saline (PBS) was purchased from Biological Industries (Beit Haemek, Israel).

Primers targeting the H1N1 nucleoprotein gene and mouse β-actin were synthesized by Sangon Biotech (Shanghai) Co., Ltd. (Shanghai, China). Isoflurane was obtained from Shenzhen RWD Life Science Co., Ltd. (Shenzhen, Guangdong, China). Tryptone was purchased from Thermo Fisher Scientific Inc. (Waltham, MA, USA), whereas yeast extract and agar were purchased from Biofroxx GmbH (Hohenhameln, Germany). Paraformaldehyde was obtained from Beijing Lanjieke Technology Co., Ltd. (Beijing, China). All other reagents were of analytical grade.

### 2.2. Animals

Specific pathogen-free female ICR mice (3–4 weeks old; 18–20 g) were purchased from Jinan Pengyue Experimental Animal Breeding Co., Ltd. (Jinan, Shandong, China). Three mice were housed per cage at 20–26 °C and 40–70% relative humidity under a 12 h/12 h light/dark cycle, with ad libitum access to standard laboratory chow and water.

All animal procedures were conducted in accordance with institutional guidelines for the care and use of laboratory animals. The study protocol was reviewed and approved by the Animal Ethics Committee of Shandong University of Traditional Chinese Medicine.

### 2.3. Color Measurement

The LJF samples were milled and passed through a No. 9 sieve. The color of each powdered sample was measured using a CS-826 benchtop spectrophotometer (CaiPu Technology (Zhejiang) Co., Ltd., Hangzhou, Zhejiang, China) and expressed in the CIELAB color space. L* denotes lightness from 0 (black) to 100 (white); positive and negative a* values indicate redness and greenness, respectively, whereas positive and negative b* values indicate yellowness and blueness, respectively. Principal component analysis (PCA) was performed on the colorimetric data using a microbial bioinformatics platform.

### 2.4. HS–GC–IMS Analysis

Volatile profiles were analyzed using a headspace gas chromatography–ion mobility spectrometry system (HS–GC–IMS; FlavourSpec^®^, G.A.S., Dortmund, Germany). A 1.5 g portion of LJF powder was transferred to a 20 mL headspace vial and incubated at 80 °C for 30 min with agitation at 500 rpm. The syringe temperature was maintained at 85 °C, and 500 μL of headspace gas was injected for analysis.

Chromatographic separation was performed on a highly polar MXT-WAX capillary column (15 m × 0.53 mm, 1.0 μm; Restek, Bellefonte, PA, USA) maintained at 60 °C. The IMS detector was maintained at 45 °C. High-purity nitrogen (≥99.999%) was used as both the carrier and drift gas, with the drift-gas flow rate set at 75 mL/min. The carrier-gas flow rate was initially 2 mL/min, increased linearly to 10 mL/min over 0–5 min, to 75 mL/min over 5–20 min, and to 150 mL/min over 20–30 min. All samples were independently analyzed in triplicate.

Tentative compound assignment was performed using C4–C9 n-ketones as calibration standards for retention-index calibration. Volatile ion signals were characterized by their retention index (RI), retention time (Rt), and drift time (Dt) and were tentatively assigned by comparison with entries in the software-embedded NIST 2020 and IMS Library 1.5 databases. Signals for which no acceptable library match was obtained were reported as unidentified. Monomer and dimer ion signals originating from the same chemical entity were reported separately as ion signals but counted as a single unique VOC. Data processing was performed using the Reporter and Gallery Plot plugins to generate three-dimensional spectra, two-dimensional topographic plots, differential spectra, and fingerprint profiles for visual comparison among samples. Prior to multivariate statistical analysis, the VOC peak-area data were subjected to Z-score normalization using SPSS, whereby each variable was mean-centered and scaled by its standard deviation. The resulting standardized VOC signal matrix was subsequently imported into SIMCA 14.1 for OPLS–DA. Model performance was evaluated using R^2^Y, Q^2^, and a 200-permutation test, and variables with VIP scores greater than 1 were retained as candidate discriminant signals.

### 2.5. Assessment of Protective Effects Against Secondary Bacterial Infection

#### 2.5.1. Preparation of the LJF Extract for Oral Administration

According to the Pharmacopoeia of the People’s Republic of China, the maximum recommended daily dose of LJF for adults is 15 g. The corresponding mouse-equivalent dose was calculated using the body-size conversion formula *d_B_* = *d_A_* × (*R_B_*/*R_A_*) × (*W_A_*/*W_B_*)^1/3^ [14]. In the present calculation, *d_A_* = 0.25 g/kg/day, *R_B_*/*R_A_* = 5.9, and (*W_A_*/*W_B_*)^1/3^ = 1.82. Accordingly, the mouse-equivalent dose was calculated as 0.25 × 5.9 × 1.82 = 2.68 g/kg/day. For aqueous extraction, 100 g of LJF decoction pieces were immersed in 20 volumes (*v*/*w*) of water for 30 min and then boiled for 20 min. The filtrate was collected, and the residual material was re-extracted with 15 volumes (*v*/*w*) of boiling water for 15 min. The two filtrates were combined, concentrated under reduced pressure to 300 mL, and lyophilized to yield the LJF aqueous extract, with an average extraction yield of 39.73%. The lyophilized powder was stored at −20 °C until use and equilibrated to room temperature before oral administration. Amounts of lyophilized extract equivalent to 7.46, 14.92, and 29.84 g of crude LJF were dissolved separately in 35 mL of purified water and sonicated for 30 min to prepare the low-, medium-, and high-dose dosing solutions, respectively. Oseltamivir phosphate granules were dissolved in distilled water and administered at 11 mg/kg/day as the positive control.

#### 2.5.2. Dose Optimization of the LJF Extract Against Secondary Bacterial Infection

After a 7-day acclimation period, specific pathogen-free female ICR mice (3–4 weeks old; 18–20 g) were randomly assigned to six groups using a random number table method: control group, model group, oseltamivir phosphate (OP), low-dose LJF extract, medium-dose LJF extract, and high-dose LJF extract. Each group comprised six mice.

On day 0, the mice were anesthetized with inhaled isoflurane. Mice in the control group received 20 μL of sterile saline intranasally, whereas mice in all other groups were intranasally inoculated with 20 μL of influenza A virus A/PR/8/34 (H1N1; PR8 strain) at 0.2 LD_50_ per mouse. After inoculation, the animals were housed by treatment group and provided food and water ad libitum.

On day 3, 2 h before the *S. aureus* challenge, the low-, medium-, and high-dose groups received LJF extract by oral gavage at 2.68, 5.36, and 10.72 g/kg/day, respectively. The OP group received oseltamivir phosphate at 11 mg/kg/day, corresponding to the human-equivalent dose, whereas the control and model groups received an equivalent volume of distilled water.

Two hours after treatment, mice in the control group received 20 μL of sterile saline intranasally, whereas mice in the other groups were intranasally challenged with 20 μL of an *S. aureus* suspension containing 2 × 10^3^ CFU. This procedure established a model of *S. aureus* superinfection following influenza A virus infection. The assigned treatments were then continued once daily by oral gavage for five consecutive days. General clinical signs and body weight were recorded daily.

#### 2.5.3. Comparative Effects of LJF Aqueous Extracts from Different Geographical Origins Against Secondary Bacterial Infection

After a 7-day acclimation period, specific pathogen-free female ICR mice (3–4 weeks old; 18–20 g) were randomly allocated to the control group, model group, oseltamivir phosphate (OP) group, and 27 LJF treatment groups corresponding to samples S1–S27. Each group comprised three mice.

On day 0, the mice were anesthetized with inhaled isoflurane. Mice in the control group received 20 μL of sterile saline intranasally, whereas mice in all other groups were intranasally inoculated with 20 μL of influenza A virus A/PR/8/34 (H1N1; PR8 strain) at 0.2 LD_50_ per mouse. After inoculation, the animals were housed by treatment group and provided food and water ad libitum.

On day 3, 2 h before the *S. aureus* challenge, mice in groups S1–S27 received the corresponding LJF aqueous extracts by oral gavage at 10.72 g/kg/day, equivalent to four times the human-equivalent dose. The OP group received oseltamivir phosphate at 11 mg/kg/day, whereas the control and model groups received an equivalent volume of distilled water.

Two hours after treatment, mice in the control group received 20 μL of sterile saline intranasally, whereas mice in the other groups were intranasally challenged with 20 μL of an *S. aureus* suspension containing 2 × 10^3^ CFU. The assigned treatments were subsequently continued once daily by oral gavage for five consecutive days.

#### 2.5.4. Sample Collection

At the designated endpoint, the mice were deeply anesthetized with isoflurane and euthanized before necropsy. The lungs were immediately excised and weighed. The left upper lung lobe was immersed in neutral paraformaldehyde fixative for histological examination, whereas the remaining lung tissue was divided into aliquots, snap-frozen in liquid nitrogen, and stored at −80 °C until analysis.

#### 2.5.5. Determination of Pulmonary Bacterial Burden by Plate Counting

The right upper lung lobe was accurately weighed and thoroughly homogenized in sterile PBS at a tissue-to-buffer ratio of 1:10 (*w*/*v*). The resulting lung homogenate was serially diluted with sterile PBS to final dilutions of 10^−6^–10^−7^. Subsequently, 100 μL of the diluted suspension was evenly spread onto LB agar plates composed of agar, tryptone, sodium chloride, yeast extract, and double-distilled water at a ratio of 3:2:2:1:200. The plates were incubated at 37 °C for 24 h, after which bacterial colonies were counted. Pulmonary bacterial burden was calculated and expressed as colony-forming units per gram of lung tissue (CFU/g).

#### 2.5.6. Histopathological Examination of Lung Tissue

The fixed left upper lung lobe was paraffin-embedded, sectioned, and stained with hematoxylin and eosin. Histopathological changes were examined and documented by light microscopy for qualitative assessment of pulmonary lesions.

#### 2.5.7. Quantitative Real-Time PCR Analysis

Total RNA was extracted from mouse lung tissue using a commercial total RNA extraction kit according to the manufacturer’s instructions. Complementary DNA was synthesized using the PrimeScript RT Reagent Kit (Takara Bio Inc., Shiga, Japan), and quantitative real-time PCR was performed using SYBR Premix Ex Taq II (Takara Bio Inc.) according to the manufacturer’s protocol.

The amplification program comprised an initial denaturation at 95 °C for 30 s, followed by 40 cycles at 95 °C for 5 s and 60 °C for 30 s. Cycle threshold (Ct) values were recorded, and the amplification and melting curves were examined to assess reaction validity. Relative H1N1-NP RNA expression was normalized to β-actin and calculated using the 2^−ΔΔCt^ method. The primer sequences are provided in Table 1.

#### 2.5.8. Calculation of Pharmacodynamic Improvement Rates

For the 27-batch comparison, improvement rates were calculated from the corresponding group means (n = 3 mice per LJF batch) using the following equations:

Lung index improvement rate (%) = [(mean lung index_model − mean lung index_treatment)/(mean lung index_model − mean lung index_control)] × 100.

Pulmonary bacterial burden improvement rate (%) = [(mean bacterial burden_model − mean bacterial burden_treatment)/(mean bacterial burden_model − mean bacterial burden_control)] × 100.

Relative NP RNA expression improvement rate (%) = [(mean NP RNA_model − mean NP RNA_treatment)/(mean NP RNA_model − mean NP RNA_control)] × 100.

### 2.6. Statistical Analysis

Statistical analyses were performed using SPSS version 26.0, and figures were generated using GraphPad Prism version 9.0. Quantitative data are presented as the mean ± standard deviation. For the pharmacodynamic data, normality was assessed using the Shapiro–Wilk test, and homogeneity of variance was evaluated using Levene’s test before group comparisons. For the dose-optimization experiment, the lung index, pulmonary bacterial burden, and relative NP RNA expression were analyzed by one-way analysis of variance (ANOVA). When the overall ANOVA indicated a significant difference, pairwise comparisons were performed using the least significant difference (LSD) test for data with homogeneous variances or Dunnett’s T3 test for data with heterogeneous variances. Body-weight changes were evaluated descriptively to monitor the general condition of the animals.

For the comparison of the 27 LJF batches from different geographical origins, each individual LJF batch was treated as the analytical unit, with nine independent batches per geographical region. Regional comparisons of the lung-index improvement rate and relative NP RNA-expression improvement rate were performed using one-way ANOVA, followed by LSD or Dunnett’s T3 pairwise comparisons according to the result of Levene’s test. A *p* value < 0.05 was considered statistically significant.

Colorimetric data were analyzed by principal component analysis as described in Section 2.3. HS–GC–IMS fingerprint data and the multivariate analyses were processed and evaluated as described in Section 2.4, including OPLS–DA, 200-permutation testing, and VIP analysis.

## 3. Results

### 3.1. Appearance Characteristics of LJF from Different Geographical Origins

The CIELAB color parameters of LJF samples from Shandong, Henan, and Hebei are summarized in Supplementary Table S2. Differences were observed in L*, a*, and b* values among the three geographical origins. Shandong samples generally exhibited higher L* and b* values, whereas Hebei samples showed lower L* values. Several Henan and Hebei samples had negative a* values, indicating a slight shift toward the green region of the CIELAB color space.

PCA based on the standardized CIELAB parameters further demonstrated differences in color characteristics among the three regions (Supplementary Figure S1). PC1 and PC2 explained 57.6% and 34.9% of the total variance, respectively, accounting for 92.5% of the cumulative variance. Shandong samples were relatively well separated from most Henan and Hebei samples, whereas the latter two groups showed partial overlap. These results indicate origin-associated variation in the color characteristics of LJF.

### 3.2. GC–IMS Spectral Analysis

**Figure 1:**
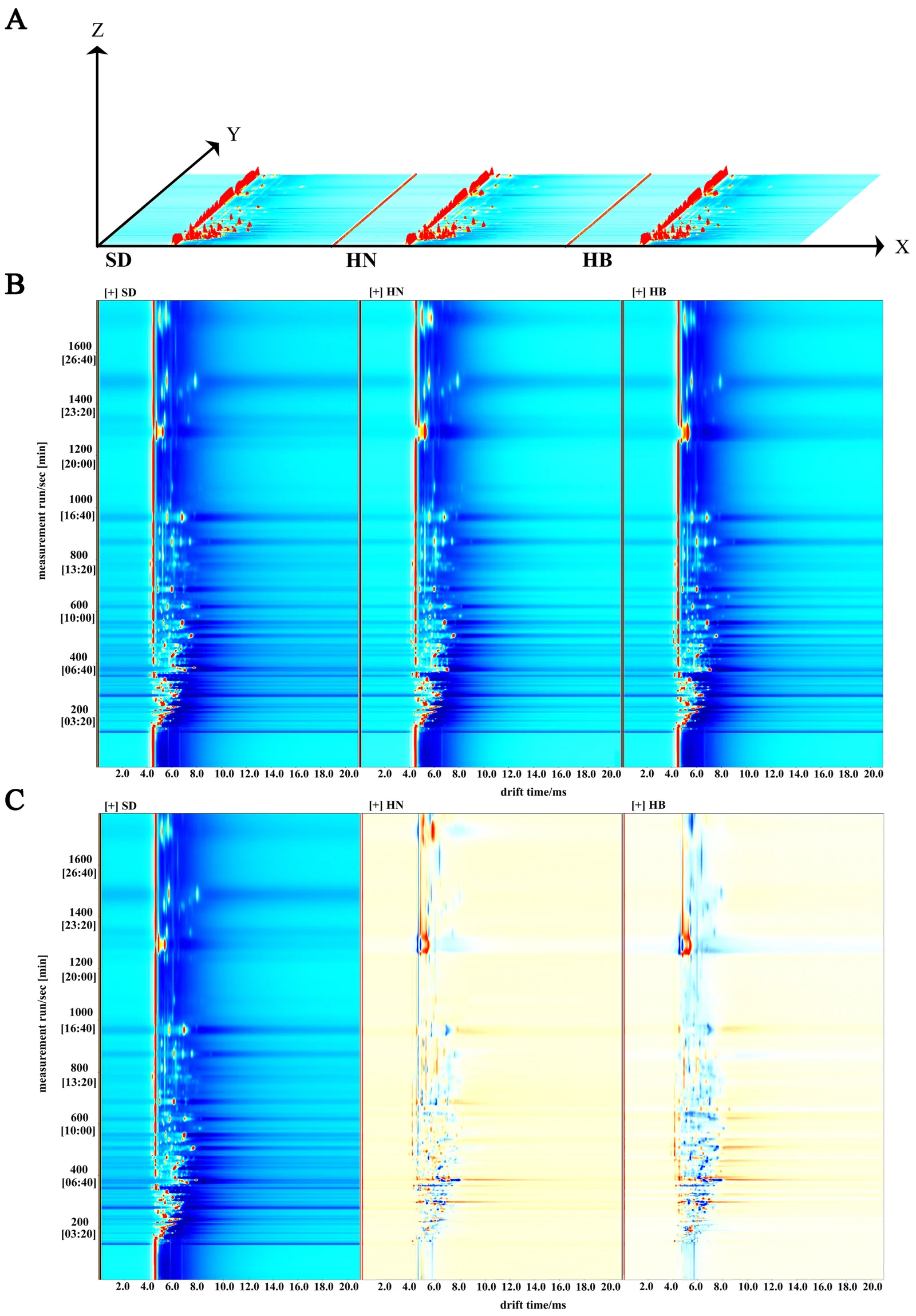
HS–GC–IMS spectra and differential plots of volatile organic compounds in LJF samples from different geographical origins: (**A**) three-dimensional spectra; (**B**) two-dimensional topographic plots; (**C**) two-dimensional spectra and corresponding differential plots.

HS–GC–IMS was used to compare the volatile profiles of LJF samples from Shandong, Henan, and Hebei because of its rapid separation and sensitive detection of VOCs with minimal sample preparation [15]. The resulting data were visualized as three-dimensional spectra, two-dimensional topographic plots, and differential spectra (Figure 1).

The three-dimensional and two-dimensional profiles showed broadly similar overall distribution patterns among samples from the three geographical origins, indicating that the general VOC profiles of LJF were comparable across regions. Nevertheless, clear differences were observed in the number and relative intensities of individual signals (Figure 1A,B). In the topographic plots, stronger signal responses were represented by a gradual color transition from blue to red [8,16,17]. These observations suggest that geographical variation was reflected primarily in the relative distribution and abundance of specific volatile features rather than in major changes in the overall spectral pattern.

The differential spectra further highlighted regional variation in VOC profiles (Figure 1C). In the comparison between Shandong (SD) and Henan (HN), signals with higher relative intensities in SD were mainly distributed at drift times of 4.0–12.0 ms and retention times of 200–800 s. In contrast, the SD–Hebei (HB) comparison showed several signals with lower relative intensities in SD, particularly at drift times of 6.0–14.0 ms and retention times of 400–1000 s [18]. These contrasting patterns indicate that geographical differences involved specific subsets of volatile signals rather than a uniform increase or decrease in the overall volatile profile. At longer retention times of approximately 1000–1600 s, the signal patterns were comparatively similar among the three regional groups. Overall, the major origin-associated differences in the volatile profiles of LJF were concentrated primarily within the intermediate retention-time region. These spectral differences provided the basis for subsequent fingerprint analysis and multivariate discrimination of LJF samples according to geographical origin.

### 3.3. Tentative Identification of Volatile Compounds in LJF from Different Geographical Origins

A total of 62 volatile ion signals were detected across the LJF samples. Of these, 49 signals were tentatively assigned to 36 unique VOCs, whereas 13 signals remained unidentified. The 36 tentatively assigned VOCs comprised 11 ketones, 6 alcohols, 6 aldehydes, 7 esters, 1 acid, 1 furan, and 4 compounds from other chemical classes. Detailed assignment information and signal characteristics are presented in Table 2. Among the tentatively assigned VOCs, ketones, alcohols, aldehydes, and esters were the predominant chemical classes.

### 3.4. Fingerprint Analysis of Volatile Compounds

**Figure 2:**
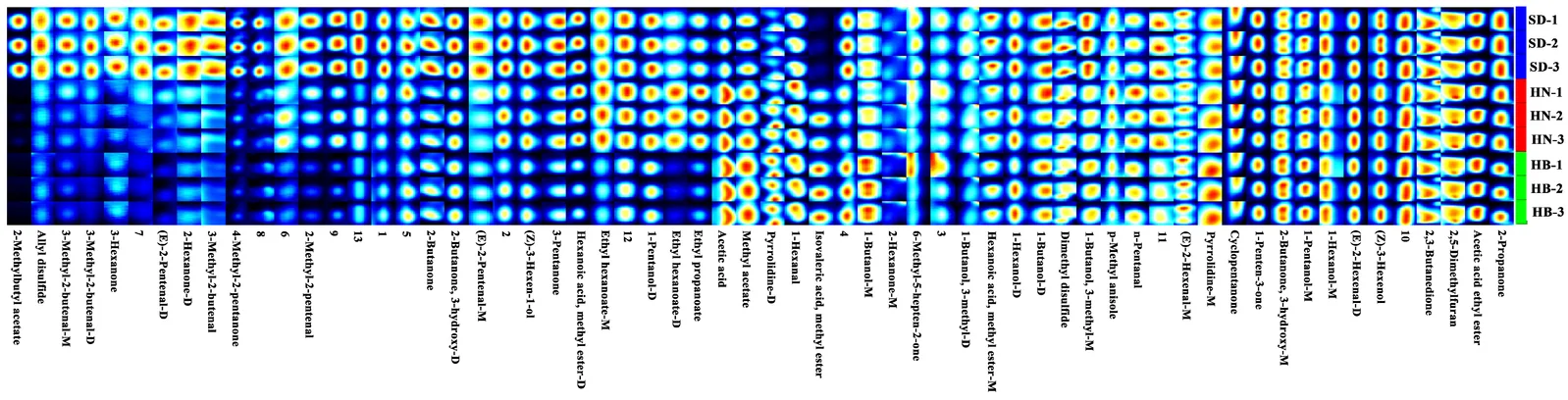
GC–IMS fingerprint profiles of LJF samples from different geographical origins.

The GC–IMS fingerprint profiles revealed distinct origin-associated differences in the volatile composition of LJF (Figure 2). Each colored cell represents the signal intensity of a volatile feature; cells within the same column correspond to the same feature across samples, whereas each row represents the volatile profile of an individual sample. Color intensity reflects relative signal abundance and enables rapid visual comparison among samples [19]. Samples within each geographical group exhibited broadly similar fingerprint patterns, while the relative intensities of multiple volatile signals differed among the Shandong (SD), Henan (HN), and Hebei (HB) groups.

A group of volatile signals showed relatively higher intensities in SD samples than in HN and HB samples, including 2-methylbutyl acetate, allyl disulfide, 3-methyl-2-butenal monomer and dimer, 3-hexanone, 2-hexanone dimer, 3-methyl-2-butanol, 4-methyl-2-pentanone, and unidentified signals 7 and 8. These features may contribute to the characteristic volatile fingerprint of LJF from Shandong. In contrast, several signals in other regions of the fingerprint were relatively more intense in HN or HB samples, indicating that geographical differences involved multiple volatile features rather than a uniform shift in overall signal abundance.

The HN and HB samples showed more similar fingerprint patterns to each other than either did to the SD samples, although differences in individual signal intensities remained evident. Overall, the fingerprint analysis demonstrated differences in the relative distribution of volatile signals among the three geographical groups and provided a basis for subsequent multivariate discrimination.

### 3.5. OPLS–DA Analysis

OPLS–DA was applied to the VOC signal matrix to assess supervised discrimination among LJF samples from different geographical origins. This approach separates variation associated with predefined groups from orthogonal variation unrelated to classification and is widely used in food quality assessment and geographical differentiation [20,21].

Model performance was evaluated using R^2^Y, which describes the goodness of fit for the response variable, and Q^2^, which estimates the cross-validated predictive ability of the model. Values approaching 1 indicate stronger model fit and predictive performance, and Q^2^ values greater than 0.5 are generally considered acceptable [22].

**Figure 3:**
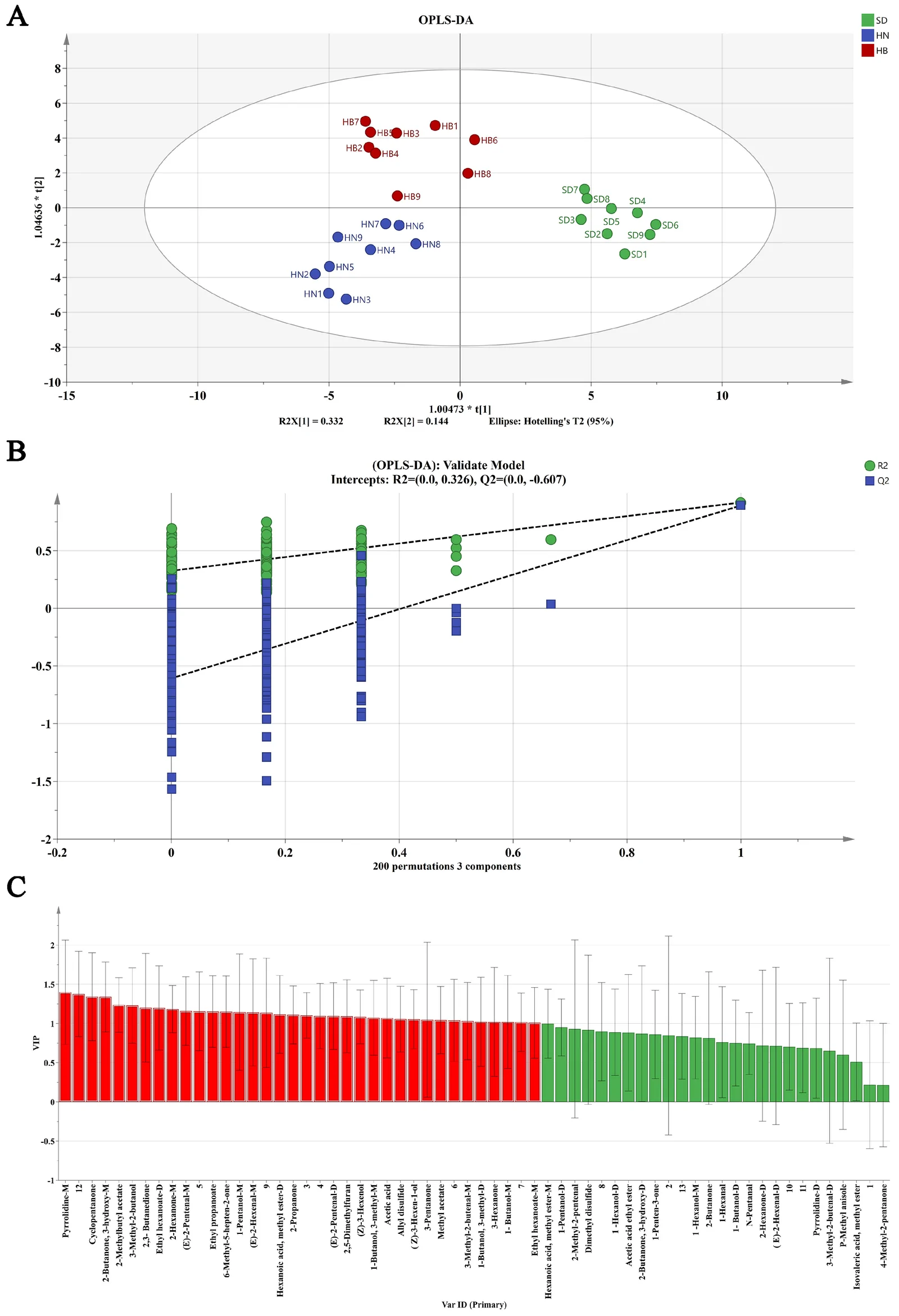
OPLS–DA of VOC profiles in LJF samples from different geographical origins: (**A**) score plot; (**B**) 200-permutation test; (**C**) VIP score distribution showing the variables contributing most strongly to sample discrimination.

The OPLS–DA score plot showed the clearest separation of Shandong samples from those from Henan and Hebei, indicating a more distinct volatile profile for the Shandong group. Henan and Hebei samples clustered more closely, suggesting greater similarity between these two regions (Figure 3A). The model yielded R^2^Y = 0.898 and Q^2^ = 0.844, supporting satisfactory model fit and overall group separation.

Model robustness was further assessed using 200 permutation tests. The *y*-axis intercepts of the R^2^ and Q^2^ regression lines were 0.326 and −0.607, respectively. The permuted R^2^ and Q^2^ values were lower than the corresponding values for the original model, and the Q^2^ regression line had a negative intercept. These results indicated no evident overfitting and supported the stability of the model (Figure 3B) [23,24].

VIP scores were used to estimate the contribution of individual variables to sample discrimination. Thirty-two volatile signals with VIP scores greater than 1 were retained as candidate discriminant variables (Figure 3C; Supplementary Table S3) [25]. Representative high-VIP signals included compound 12, cyclopentanone, 3-hydroxy-2-butanone monomer, 2-methylbutyl acetate, 3-methyl-2-butanol, 2,3-butanedione, ethyl hexanoate dimer, and 2-hexanone monomer. These variables contributed to regional separation but require confirmation in independent sample sets before they can be considered validated geographical markers.

### 3.6. Dose Optimization of the LJF Aqueous Extract in the Secondary Bacterial Infection Model

**Figure 4:**
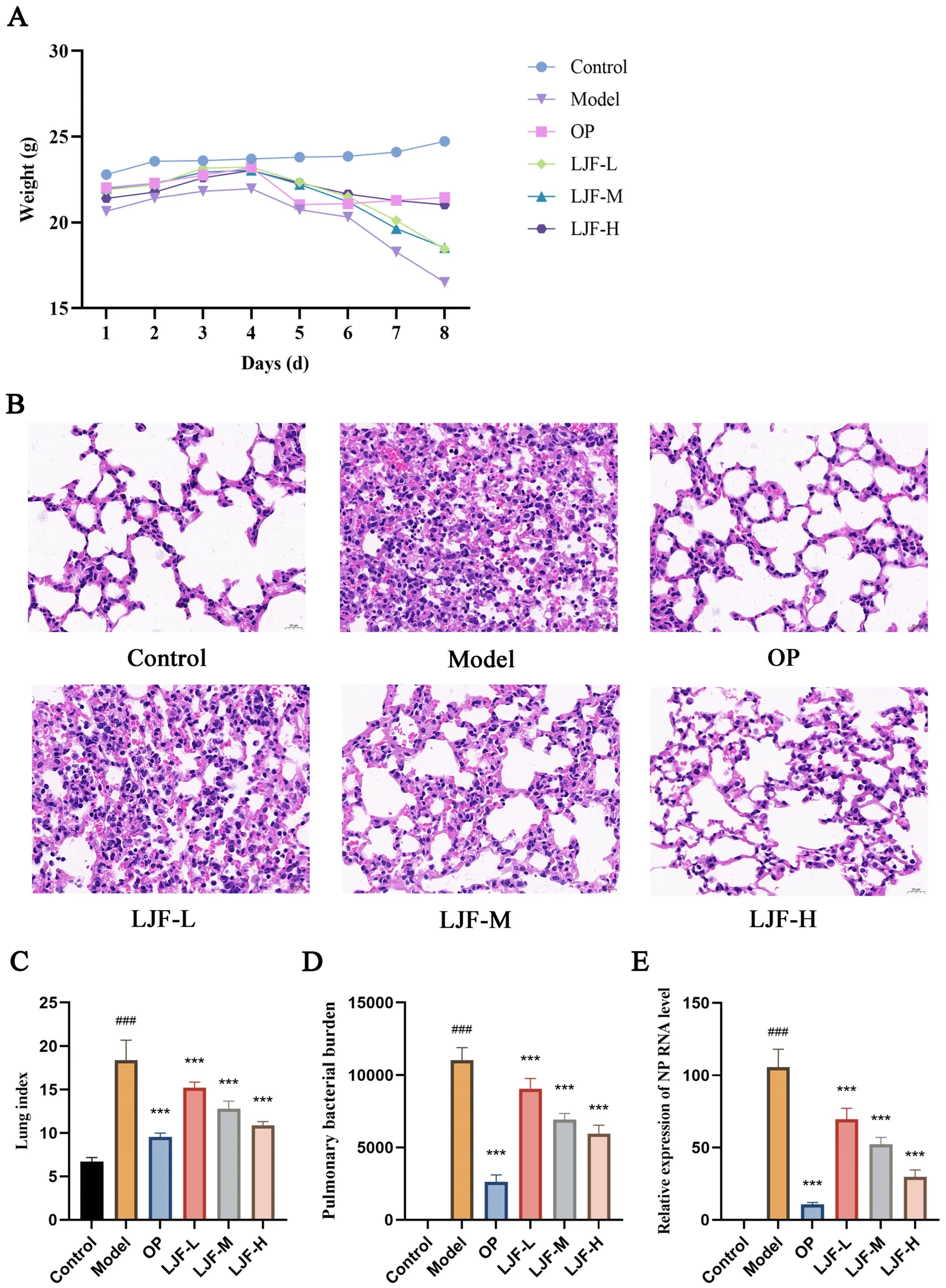
Protective effects of LJF aqueous extract in the secondary bacterial infection model: (**A**) changes in body weight; (**B**) representative hematoxylin and eosin-stained lung sections (original magnification, ×400); (**C**) lung index; (**D**) pulmonary bacterial burden; (**E**) relative expression of NP RNA level. Data are presented as the mean ± SD (n = 6). ^###^ *p* < 0.001 versus the control group; *** *p* < 0.001 versus the model group.

In the secondary bacterial infection model (Figure 4A), mice in the model group developed lethargy and reduced food intake following the *S. aureus* challenge. Body weight began to decline on day 4, corresponding to the first day after bacterial challenge. In the OP group, body weight began to recover from day 5, whereas it continued to decline in the model group. LJF treatment attenuated infection-associated weight loss to varying degrees, with smaller mean reductions observed at higher doses. Among the LJF-treated groups, the high-dose group showed the least body weight loss and a trajectory similar to that of the OP group.

Histopathological examination revealed distinct differences in pulmonary morphology among the groups (Figure 4B). Lung tissues from the control group showed intact alveolar and bronchial structures without apparent pathological abnormalities. In contrast, the model group exhibited focal alveolar septal thickening, inflammatory-cell infiltration, and structural damage around the alveoli and blood vessels. Similar alterations were observed in the low-dose LJF group, whereas these changes were less pronounced in the medium-dose group, although localized alveolar epithelial proliferation remained evident. The high-dose group showed comparatively limited inflammatory-cell infiltration and better preservation of alveolar architecture, with pulmonary morphology resembling that observed in the OP group.

Compared with the control group, the model group showed significantly increased lung index, pulmonary bacterial burden, and relative NP RNA expression (*p* < 0.001). All three endpoints were significantly reduced in the LJF-treated groups compared with the model group (*p* < 0.001) (Figure 4C–E). Progressively lower mean values were observed across the low-, medium-, and high-dose LJF groups, with the high-dose group showing the greatest overall improvement among the three treatment groups. Based on the combined pharmacodynamic outcomes, the high dose of 10.72 g crude LJF equivalents/kg/day was selected for the subsequent comparison of LJF samples from different geographical origins.

### 3.7. Comparative Protective Effects of LJF Aqueous Extracts from Different Geographical Origins Against Secondary Bacterial Infection

The pharmacodynamic outcomes of the 27 LJF batches are summarized in Table 3. Across all batches, improvement rates ranged from 28.13% to 56.69% for the lung index, from 27.92% to 62.15% for pulmonary bacterial burden, and from 51.54% to 72.14% for relative NP RNA expression.

The mean lung index improvement rates were 52.62 ± 3.04%, 43.55 ± 4.75%, and 31.28 ± 2.47% for samples from Shandong, Henan, and Hebei, respectively. Pairwise comparisons showed that the improvement rate was higher in Shandong than in Henan and Hebei and was also higher in Henan than in Hebei (*p* < 0.001).

For pulmonary bacterial burden, the mean improvement rates were 39.48 ± 5.05% for Shandong, 41.03 ± 8.44% for Henan, and 32.66 ± 4.09% for Hebei. Pairwise comparisons showed higher improvement rates in both Shandong and Henan than in Hebei (*p* < 0.05), whereas no significant difference was observed between Shandong and Henan.

The mean improvement rates for relative NP RNA expression were 68.30 ± 2.65%, 65.24 ± 4.33%, and 55.78 ± 2.73% for Shandong, Henan, and Hebei, respectively. Both Shandong and Henan samples showed higher improvement rates than Hebei samples (*p* < 0.001), whereas the difference between Shandong and Henan was not significant.

Collectively, these results demonstrated origin-associated differences in the pharmacodynamic performance of LJF. Samples from Shandong and Henan generally showed greater improvement across the evaluated endpoints than those from Hebei, although the pattern of regional differences varied among individual pharmacodynamic parameters.

## 4. Conclusions

This study integrated HS–GC–IMS profiling, multivariate analysis, and in vivo pharmacodynamic assessment to characterize origin-associated quality variation in LJF from Shandong, Henan, and Hebei. A total of 62 volatile ion signals were detected, of which 49 were tentatively assigned to 36 unique VOCs, while 13 remained unidentified. Ketones, alcohols, aldehydes, and esters represented the predominant chemical classes among the tentatively assigned VOCs. Fingerprint analysis and OPLS–DA revealed clear geographical variation in the volatile profiles, with Shandong samples showing the most distinct separation from those from Henan and Hebei. In addition, 32 signals with VIP scores greater than 1 were retained as candidate discriminant variables contributing to geographical differentiation. These variables, however, require further confirmation and independent validation before they can be regarded as reliable geographical markers.

In the influenza-associated *S. aureus* superinfection model, LJF aqueous extracts reduced the lung index, pulmonary bacterial burden, and relative NP RNA expression. Pharmacodynamic performance also varied among geographical origins, with samples from Shandong and Henan generally showing greater improvement than those from Hebei across the evaluated endpoints. Together, the chemical and pharmacodynamic datasets demonstrated parallel origin-associated variation within the same set of LJF samples. However, direct relationships between specific volatile compounds and pharmacodynamic effects were not established. The two datasets therefore provide complementary evidence for origin-related quality differences in LJF.

Importantly, compound assignments obtained by HS–GC–IMS were based on chromatographic and ion-mobility characteristics, including retention index, retention time, and drift time, matched against reference libraries. These assignments should therefore be regarded as tentative library-based assignments rather than unambiguous structural identifications. Future studies incorporating authentic reference standards and complementary GC–MS analysis are warranted to confirm the identities of key discriminatory VOCs. Further validation using larger and independent sample sets, together with targeted chemical–efficacy correlation or spectrum–effect analyses, will also be necessary to assess the robustness of candidate geographical markers and to clarify the contribution of specific volatile constituents to pharmacodynamic variation. With further refinement and validation, this strategy may have broader potential for origin-related quality evaluation and traceability studies of other medicinal and edible herbal materials.

## Figures and Tables

**Table 1:** Primer sequences used for qRT-PCR detection.

Name	Direction	Sequence (5′–3′)
Mouse H1N1-NP	For	GTATGGACCTGCCGTAGC
	Rev	CGTCTACCTGCAGCCTC
Mouse β-Actin	For	CATTGCTGACAGGATGCAGAAGG
	Rev	TGCTGGAAGGTGGACAGTGAGG

**Table 2:** Tentative library assignments of volatile ion signals detected in LJF by HS–GC–IMS.

Category	Compound	CAS#	Formula	MW	RI	Rt/s	Dt/ms	Comment
Ketones	6-Methyl-5-hepten-2-one	C110930	C_8_H_14_O	126.2	1347.5	812.67	1.17713	
	2-Butanone, 3-hydroxy-M	C513860	C_4_H_8_O_2_	88.1	1296.3	687.759	1.08063	Monomer
	2-Butanone, 3-hydroxy-D	C513860	C_4_H_8_O_2_	88.1	1297.7	690.941	1.33503	Dimer
	Cyclopentanone	C120923	C_5_H_8_O	84.1	1138.9	426.346	1.31803	
	3-Hexanone	C589388	C_6_H_12_O	100.2	1067.4	342.421	1.45545	
	1-Penten-3-one	C1629589	C_5_H_8_O	84.1	1041.5	316.641	1.30916	
	2-Hexanone-M	C591786	C_6_H_12_O	100.2	1052.5	327.396	1.20788	Monomer
	2-Hexanone-D	C591786	C_6_H_12_O	100.2	1054.1	328.889	1.50482	Dimer
	4-Methyl-2-pentanone	C108101	C_6_H_12_O	100.2	1017.5	294.64	1.1739	
	3-Pentanone	C96220	C_5_H_10_O	86.1	996.6	276.866	1.35948	
	2,3-Butanedione	C431038	C_4_H_6_O_2_	86.1	940.5	242.909	1.19659	
	2-Butanone	C78933	C_4_H_8_O	72.1	908.8	225.662	1.25427	
	2-Propanone	C67641	C_3_H_6_O	58.1	829.1	187.37	1.11512	
Alcohols	(Z)-3-Hexenol	C928961	C_6_H_12_O	100.2	1399.7	963.04	1.24555	Monomer
	(Z)-3-Hexen-1-ol	C928961	C_6_H_12_O	100.2	1400.2	964.631	1.51048	Dimer
	1-Hexanol-M	C111273	C_6_H_14_O	102.2	1369.9	873.932	1.33503	Monomer
	1-Hexanol-D	C111273	C_6_H_14_O	102.2	1369.6	873.136	1.64734	Dimer
	1-Pentanol-M	C71410	C_5_H_12_O	88.1	1262.1	620.344	1.259	Monomer
	1-Pentanol-D	C71410	C_5_H_12_O	88.1	1263	622.094	1.52017	Dimer
	1-Butanol, 3-methyl-M	C123513	C_5_H_12_O	88.1	1217.2	542.427	1.23975	Monomer
	1-Butanol, 3-methyl-D	C123513	C_5_H_12_O	88.1	1217.2	542.427	1.48718	Dimer
	1-Butanol-M	C71363	C_4_H_10_O	74.1	1155.7	449.341	1.18406	Monomer
	1-Butanol-D	C71363	C_4_H_10_O	74.1	1155.4	448.818	1.38553	Dimer
	3-Methyl-2-butanol	C598754	C_5_H_12_O	88.1	1104.2	382.813	1.42964	
Aldehydes	(E)-2-Hexenal-M	C6728263	C_6_H_10_O	98.1	1232.5	567.815	1.18202	Monomer
	(E)-2-Hexenal-D	C6728263	C_6_H_10_O	98.1	1227.8	559.936	1.51742	Dimer
	3-Methyl-2-butenal-M	C107868	C_5_H_8_O	84.1	1209.6	530.17	1.09542	Monomer
	3-Methyl-2-butenal-D	C107868	C_5_H_8_O	84.1	1210.7	531.921	1.35797	Dimer
	2-Methyl-2-pentenal	C623369	C_6_H_10_O	98.1	1160.3	455.755	1.48993	
	(E)-2-Pentenal-M	C1576870	C_5_H_8_O	84.1	1124.8	408.068	1.11364	Monomer
	(E)-2-Pentenal-D	C1576870	C_5_H_8_O	84.1	1125.2	408.628	1.36526	Dimer
	1-Hexanal	C66251	C_6_H_12_O	100.2	1097.2	374.501	1.56368	
	n-Pentanal	C110623	C_5_H_10_O	86.1	1003.1	282.094	1.43487	
Esters	Ethyl hexanoate-M	C123660	C_8_H_16_O_2_	144.2	1242.6	585.325	1.34422	Monomer
	Ethyl hexanoate-D	C123660	C_8_H_16_O_2_	144.2	1242.6	585.325	1.79509	Dimer
	Hexanoic acid, methyl ester-M	C106707	C_7_H_14_O_2_	130.2	1196.6	510.034	1.28924	Monomer
	Hexanoic acid, methyl ester-D	C106707	C_7_H_14_O_2_	130.2	1196.6	510.034	1.68099	Dimer
	2-Methylbutyl acetate	C624419	C_7_H_14_O_2_	130.2	1105.8	384.725	1.72837	
	Isovaleric acid, methyl ester	C556241	C_6_H_12_O_2_	116.2	1023.8	300.216	1.53152	
	Ethyl propanoate	C105373	C_5_H_10_O_2_	102.1	967.6	258.758	1.45109	
	Acetic acid ethyl ester	C141786	C_4_H_8_O_2_	88.1	884.2	213.076	1.33483	
	Methyl acetate	C79209	C_3_H_6_O_2_	74.1	842.9	193.499	1.19842	
Acids	Acetic acid	C64197	C_2_H_4_O_2_	60.1	1490.7	1295.255	1.16894	
Furans	2,5-Dimethylfuran	C625865	C_6_H_8_O	96.1	920.2	231.722	1.35863	
Others	Allyl disulfide	C2179579	C_6_H_10_S_2_	146.3	1478.9	1246.552	1.21059	
	Dimethyl disulfide	C624920	C_2_H_6_S_2_	94.2	1048.8	323.737	1.14429	
	Pyrrolidine-M	C123751	C_4_H_9_N	71.1	1030.3	306.141	1.07724	Monomer
	Pyrrolidine-D	C123751	C_4_H_9_N	71.1	1028.4	304.399	1.27055	Dimer
	p-Methyl anisole	C104938	C_8_H_10_O	122.2	1411.3	1000.284	1.12432	
Unknow	1	unidentified	*	*	1383.8	914.508	1.1087	
	2	unidentified	*	*	1349.3	817.443	1.11572	
	3	unidentified	*	*	1336.5	784.028	1.37889	
	4	unidentified	*	*	1312.2	724.357	1.07536	
	5	unidentified	*	*	1313.9	728.335	1.22976	
	6	unidentified	*	*	1273.6	642.23	1.20814	
	7	unidentified	*	*	1258.7	614.215	1.12291	
	8	unidentified	*	*	1170.6	470.638	1.20264	
	9	unidentified	*	*	1111.7	391.827	1.35202	
	10	unidentified	*	*	1060.9	335.723	1.20757	
	11	unidentified	*	*	978.2	265.284	1.13892	
	12	unidentified	*	*	808	178.373	1.30403	
	13	unidentified	*	*	725.8	147.274	1.23254	

MW, molecular weight; RI, retention index; Rt, retention time; Dt, drift time. Compound assignments were based on matching against the HS-GC-IMS reference library and should be regarded as tentative. Monomer (M) and dimer (D) signals represent different ion forms originating from the same compound and were not counted as separate unique volatile organic compounds (VOCs).

**Table 3:** Pharmacodynamic improvement rates of LJF samples from different geographical origins and regional comparisons.

NO.	Lung Index (%)	Pulmonary Bacterial Burden (%)	Relative Expression of NP RNA Level (%)
Control	\	\	\
Model	\	\	\
OP	81.46	73.90	87.74
SD1	50.89	42.95	67.71
SD2	52.04	28.68	63.54
SD3	53.08	43.05	67.36
SD4	53.39	43.05	67.59
SD5	51.89	34.99	72.14
SD6	46.39	37.39	66.76
SD7	56.69	39.54	69.78
SD8	52.87	44.09	68.03
SD9	56.36	41.56	71.83
SD mean ± SD	52.62 ± 3.04	39.48 ± 5.05	68.30 ± 2.65
HN1	43.49	35.88	69.39
HN2	41.34	42.07	67.16
HN3	53.34	43.08	65.49
HN4	47.92	62.15	69.72
HN5	43.22	35.75	67.73
HN6	41.20	35.12	61.76
HN7	38.89	40.42	56.50
HN8	37.89	38.53	62.10
HN9	44.66	36.26	67.27
HN mean ± SD	43.55 ± 4.75	41.03 ± 8.44	65.24 ± 4.33
HB1	30.04	38.02	55.99
HB2	33.71	36.00	55.33
HB3	31.19	35.25	57.72
HB4	31.46	28.80	59.41
HB5	29.37	29.81	52.49
HB6	29.36	31.08	51.54
HB7	36.08	29.18	56.88
HB8	32.20	37.90	53.93
HB9	28.13	27.92	58.77
HB mean ± SD	31.28 ± 2.47	32.66 ± 4.09	55.78 ± 2.73

## Data Availability

The original contributions presented in this study are included in the article/Supplementary Materials. Further inquiries can be directed to the corresponding authors.

## Supplementary Materials

The following supporting information can be downloaded at https://www.mdpi.com/article/10.3390/foods15183305/s1, Table S1. Collection information and geographical origins of the 27 LJF samples. Table S2. CIELAB color parameters of LJF samples from different geographical origins. Figure S1. PCA score plot based on standardized CIELAB parameters. Table S3. VIP scores of candidate discriminant volatile signals identified by OPLS–DA.
