# Supplementary material for: Geographical Differentiation and Quality Evaluation of Lonicerae japonicae Flos by Integrating HS–GC–IMS Volatile Profiling with In Vivo Pharmacodynamic Assessment
Source: Foods. 2026 Sep 18;15(18):3305. doi: 10.3390/foods15183305 (PMC13606345; doi:10.3390/foods15183305)
Supplement: Supplementary file 1 [file foods-15-03305-s001.zip › foods-4524054-supplementary.pdf]

Supplementary Materials

Table S1. Collection information and geographical origins of the 27 LJF samples.

NO.	Source	NO.	Source	NO.	Source
SD1	Pingyi County, Linyi, Shandong Province, China	HN1	Fengqiu County, Xinxiang, Henan Provinces, China	HB1	Julu County, Xingtai, Hebei Province, China
SD2	Pingyi County, Linyi, Shandong Province, China	HN2	Fengqiu County, Xinxiang, Henan Provinces, China	HB2	Julu County, Xingtai, Hebei Province, China
SD3	Pingyi County, Linyi, Shandong Province, China	HN3	Fengqiu County, Xinxiang, Henan Provinces, China	HB3	Baoding, Hebei Province, China
SD4	Pingyi County, Linyi, Shandong Province, China	HN4	Fengqiu County, Xinxiang, Henan Provinces, China	HB4	Julu County, Xingtai, Hebei Province, China
SD5	Pingyi County, Linyi, Shandong Province, China	HN5	Fengqiu County, Xinxiang, Henan Provinces, China	HB5	Julu County, Xingtai, Hebei Province, China
SD6	Pingyi County, Linyi, Shandong Province, China	HN6	Fengqiu County, Xinxiang, Henan Provinces, China	HB6	Julu County, Xingtai, Hebei Province, China
SD7	Pingyi County, Linyi, Shandong Province, China	HN7	Fengqiu County, Xinxiang, Henan Provinces, China	HB7	Julu County, Xingtai, Hebei Province, China
SD8	Pingyi County, Linyi, Shandong Province, China	HN8	Fengqiu County, Xinxiang, Henan Provinces, China	HB8	Julu County, Xingtai, Hebei Province, China
SD9	Pingyi County, Linyi, Shandong Province, China	HN9	Fengqiu County, Xinxiang, Henan Provinces, China	HB9	Julu County, Xingtai, Hebei Province, China

All LJF samples were oven-dried after harvest and subsequently stored until analysis.

Table S2. CIELAB color parameters of LJF samples from different geographical origins.

NO.	L* value	a* value	b* value
SD1	73.86	5.03	29.36
SD2	81.35	2.43	25.05
SD3	80.39	2.21	25.31
SD4	79.18	2.23	25.34
SD5	80.2	2.46	25.76
SD6	77.1	3.17	29.17
SD7	81.16	1.68	23.74
SD8	78.07	2.67	27.52
SD9	80.07	2.44	25.22
HN1	77.72	-1.31	21.35
HN2	77.1	-1.51	20.64
HN3	77.2	-0.46	23.08
HN4	74.37	0.73	21.35
HN5	77.14	-1.41	20.48
HN6	83.86	1.07	24.91
HN7	78.43	2.21	20.14
HN8	76.43	2.41	22.21
HN9	72.1	1.65	21.43
HB1	77.18	-1.47	21.09
HB2	69.89	1.41	22.58

HB3	73.67	-0.73	21.77
HB4	75.43	-0.83	21.34
HB5	70.54	1.21	22.1
HB6	66.86	4.55	24.99
HB7	68.38	2.04	22.73
HB8	76.29	0.86	22.41
HB9	72.63	-0.28	23.92

Figure S1. PCA score plot based on standardized CIELAB parameters.

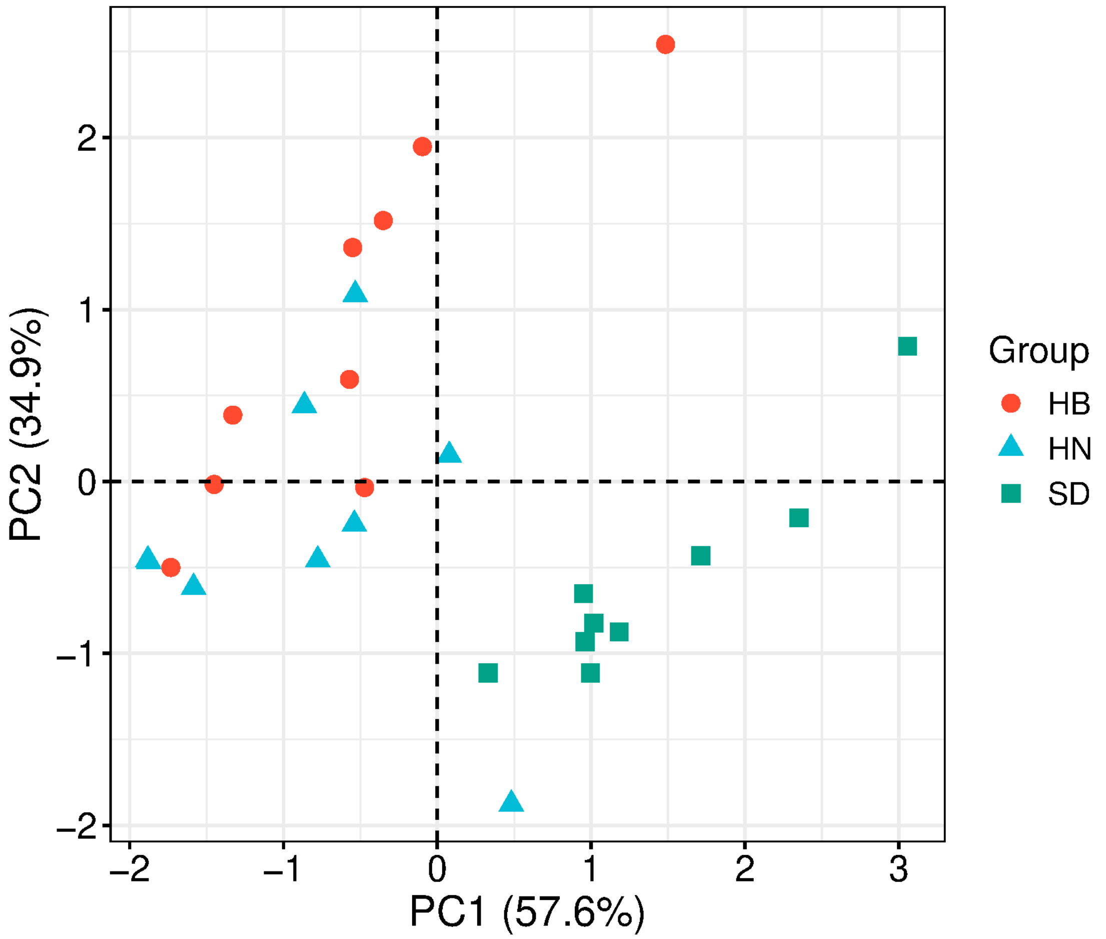

Table S3. VIP scores of candidate discriminant volatile signals identified by OPLS–DA.

NO.	Compound	VIP
1	Pyrrolidine-M	1.39924
2	12	1.37592
3	Cyclopentanone	1.34091
4	2-Butanone, 3-hydroxy-M	1.3387
5	2-Methylbutyl acetate	1.2363
6	3-Methyl-2-butanol	1.22937
7	2,3-Butanedione	1.2006
8	Ethyl hexanoate-D	1.1984

9	2-Hexanone-M	1.18388
10	(E)-2-Pentenal-M	1.15819
11	5	1.15497
12	Ethyl propanoate	1.15233
13	6-Methyl-5-hepten-2-one	1.15064
14	1-Pentanol-M	1.14423
15	(E)-2-Hexenal-M	1.14174
16	9	1.13744
17	Hexanoic acid, methyl ester-D	1.1154
18	2-Propanone	1.10989
19	3	1.10392
20	4	1.09513
21	(E)-2-Pentenal-D	1.09483
22	2,5-Dimethylfuran	1.09225
23	(Z)-3-Hexenol	1.08452
24	1-Butanol, 3-methyl-M	1.07216
25	Acetic acid	1.06871
26	Allyl disulfide	1.05575
27	(Z)-3-Hexen-1-ol	1.0539
28	3-Pentanone	1.04727
29	Methyl acetate	1.04293
30	6	1.04006
31	3-Methyl-2-butenal-M	1.03104
32	1-Butanol, 3-methyl-D	1.02156
33	3-Hexanone	1.02011
34	1-Butanol-M	1.02001
35	7	1.0161
36	Ethyl hexanoate-M	1.00854
